# Stromal Cell-Derived Factor 1α (SDF-1α) in Invasive Breast Cancer: Associations with Vasculo-Angiogenic Factors and Prognostic Significance

**DOI:** 10.3390/cancers13081952

**Published:** 2021-04-18

**Authors:** Elżbieta Zarychta, Barbara Ruszkowska-Ciastek, Kornel Bielawski, Piotr Rhone

**Affiliations:** 1 Department of Pathophysiology, Faculty of Pharmacy, Nicolaus Copernicus University, Collegium Medicum, 85-094 Bydgoszcz, Poland; ruszkowska.basia@gmail.com (B.R.-C.); kornel@doktorant.umk.pl (K.B.); 2 Clinical Ward of Breast Cancer and Reconstructive Surgery, Oncology Centre Prof. F. Łukaszczyk Memorial Hospital, 85-796 Bydgoszcz, Poland; rhonep@co.bydgoszcz.pl

**Keywords:** breast cancer, angiogenesis, SDF-1α, vascular endothelial growth factor A, VEGF-A, endothelial progenitor cells, EPCs, sVEGFR1, sVEGFR2

## Abstract

**Simple Summary:**

This prospective study was designed to examine the utility of stromal cell-derived factor 1α (SDF-1α) and selected vasculo-angiogenic parameters—vascular endothelial growth factor A (VEGF-A), the soluble forms of VEGF receptors type 1 and 2, and the number of circulating endothelial progenitor cells (circulating EPCs)—for estimating the probability of disease relapse in invasive breast cancer (IBrC) patients. Kaplan–Meier plots and log-rank and F Cox tests were used to determine the clinical outcomes in terms of disease-free survival (DFS). Patients with a combination of SDF-1α lower than 42 pg/dL and a number of circulating EPCs lower than 9.68 cells/µL had significantly shorter DFS (*p* = 0.0138); thus, the combination of these two factors shows strong diagnostic power for the prediction of disease relapse.

**Abstract:**

(1) Background: Tumour angiogenesis is critical for the progression of neoplasms. A prospective study was designed to examine the utility of stromal cell-derived factor 1α (SDF-1α) and selected vasculo-angiogenic parameters for estimating the probability of disease relapse in 84 primary, operable invasive breast cancer (IBrC) patients (40 (48%) with stage IA and 44 (52%) with stage IIA and IIB). (2) Methods: We explored the prognostic value of the plasma levels of SDF-1α, vascular endothelial growth factor A (VEGF-A), the soluble forms of VEGF receptors type 1 and 2, and the number of circulating endothelial progenitor cells (circulating EPCs) in breast cancer patients. The median follow-up duration was 58 months, with complete follow-up for the first event. (3) Results: According to ROC curve analysis, the optimal cut-off point for SDF-1α (for discriminating between patients at high and low risk of relapse) was 42 pg/mL, providing 57% sensitivity and 75% specificity. Kaplan–Meier curves for disease-free survival (DFS) showed that concentrations of SDF-1α lower than 42 pg/dL together with a VEGFR1 lower than 29.86 pg/mL were significantly associated with shorter DFS in IBrC patients (*p* = 0.0381). Patients with both SDF-1α lower than 42 pg/dL and a number of circulating EPCs lower than 9.68 cells/µL had significantly shorter DFS (*p* = 0.0138). (4) Conclusions: Our results imply the clinical usefulness of SDF-1α, sVEGFR1 and the number of circulating EPCs as prognostic markers for breast cancer in clinical settings.

## 1. Introduction

Breast cancer cases and deaths continue to rise worldwide, with invasive breast cancer (IBrC) being commonly diagnosed. Non-invasive breast carcinomas, particularly the ductal type, are not usually lethal and can often be managed by conservative approaches including breast-conserving surgery, but invasive tumours with metastatic spread remain a challenge for contemporary medicine. Breast cancer is a complex disease, which includes different subtypes with specific clinical, histopathologic and molecular features. These subtypes include: luminal A, luminal B, triple-negative (basal-like), HER2-enriched and normal-like IBrC and can be distinguished with the use of immunohistochemical (IHC) markers such as oestrogen receptor (ER), progesterone receptor (PR), expression of human epidermal growth factor receptor 2 (HER2) and expression of the proliferation marker Ki67. Intrinsic subtypes of IBrC together with traditional clinicopathological variables such as tumour size, tumour grade and nodal involvement are conventionally used for patient prognosis and management [1]. Therefore, knowledge of the molecular biology of breast cancer invasion and metastasis is crucial; identifying key molecules or signalling pathways involved in these processes should help in the development of new targeted therapy options that more efficiently prevent tumour recurrence [2].

Breast cancer invasion and metastasis are complex, multifactorial and multistep actions that involve the cooperation of many cytokines, chemokines, enzymes and growth factors derived from tumour cells and the tumour microenvironment [3]. Emerging evidence reinforces the concept that the tumour microenvironment is instrumental in tumourigenesis, particularly through the remodelling of the extracellular matrix (ECM). The degradation of the ECM by matrix metalloproteinases (MMPs) is a crucial action in tumour invasion that has a pivotal role in cancer dissemination [4]. However, the spread of cancer cells does not always lead to metastasis. It is already established that the locations of metastatic tumours depend not only on the characteristics of neoplastic cells but also on the microenvironments of specific organs. The microenvironment can support the homing of tumour cells to distinct sites with characteristic attractant molecules [5].

Chemotaxis is one of the crucial actions involved in cancer cell migration, resulting in the local invasion and systemic dissemination of a cancer. Metastasis development is organ-specific—stromal cells release signalling proteins that activate chemotaxis and attract cancer cells, resulting in the formation of metastases in particular organs [6]. Chemokines belong to a family of cytokine-like proteins that activate chemokine receptors moderating several cellular functions. One chemokine, stromal cell-derived factor-1α (SDF-1α), is present in stromal cells, including fibroblasts and endothelial cells. It binds to the transmembrane receptor CXCR4, found across a broad range of normal tissues, including the lymphatic tissues, thymus, brain, spleen, stomach and small intestine. CXCR4 is also expressed on the surfaces of T and B, hematopoietic progenitor, endothelial, endothelial progenitor, and smooth muscle cells. The receptor has also been detected on the surfaces of cancer cells, including brain, colorectal, lung, pancreas, prostate, and ovarian, leukaemia, and melanomas [7,8]. However, the expression of CXCR4 is low or non-existent in normal breast tissue but relatively high in breast cancer cells. Binding of SDF-1α to its receptor CXCR4 leads to a number of events, which are responsible for particular physiological and pathological processes including the regulation of haematopoiesis and apoptosis, immunity and mitogenic activity, cancer cell growth, migration, dissemination, and neovascularisation [2,8,9]. Moreover, the CXCL12/CXCR4 signalling pathway may be responsible for the activation of HER2 to increase invasiveness and metastasis formation in breast, oesophageal, lung, prostate and ovarian cancers [8].

Endothelial progenitor cells also play an important role in tumour development, by active interaction with cancer cells. In primary tumours, they are attracted to the tumour microenvironment, where they support tumour growth, vascularisation and metastasis. Furthermore, the cells migrate to pre-metastatic sites, where they are responsible for the formation of cellular clusters prior to the arrival of tumour cells. In the formation of the pre-metastatic niche, the SDF-1α/CXCR4 axis contributes to the recruitment of endothelial progenitors (EPCs) to the tumour microenvironment [10]. Altered levels of EPCs can be found in hepatocellular cancer, small-cell cancer, non-small-cell lung cancer, glioma, ovarian cancer, renal cell carcinoma, gastric cancer, colorectal cancer and cervical cancer. Number of EPCs was found to be useful in predicting long-term outcomes in several types of cancer. Patients with a lower number of pre-treatment EPCs had better overall survival, recurrence-free survival and response duration in small-cell lung carcinoma, non-small-cell lung carcinoma, multiple myeloma and ovarian cancer [11]. The expression of CXCR4 in malignant cells is mainly regulated by hypoxia-inducible factors 1 and 2 (HIF1 and HIF2), but it can be induced in breast cancer cells by vascular endothelial growth factor (VEGF), a well-known pro-angiogenic and pro-inflammatory molecule. The overexpression of VEGF is crucial for the development of tumour cells; VEGF promotes the development of new blood vessels and carcinogenesis through SDF-1α signalling. The role of VEGF and its receptors in metastasis formation and tumour invasiveness has been claimed in numerous cancers, such as non-small-cell lung cancer, small-cell lung cancer, mesothelioma, hepatocellular carcinoma, cholangiocarcinoma, gallbladder cancer, renal cell carcinoma, glioblastoma, melanoma, osteosarcoma, Ewing sarcoma, chondrosarcoma and pancreatic cancer [12]. The organ-specific migration of malignant cells and increase in their invasion through endothelial cells, bone marrow stromal cells and fibroblasts is directed by the activation of CXCR4 [2,9,13].

Thus, early diagnosis is very important to limit the mortality rate of neoplasms. Therefore, predictive biomarkers are the aspiration of many ongoing studies and remain sought after. The aim of our study was, therefore, to examine whether SDF-1α levels are a valuable biomarker for assessing disease relapse and monitoring disease progression in patients with invasive breast cancer (IBrC). We also investigated the association of SDF-1α with selected vasculo-angiogenic factors in terms of prognosis.

## 2. Materials and Methods

### 2.1. Patient Selection

This prospective study evaluated the value of stromal cell-derived factor 1α (SDF- 1α) for diagnosing disease relapse in 84 invasive breast cancer patients admitted to the Clinical Ward of Breast Cancer and Reconstructive Surgery, Oncology Center, Prof. F. Łukaszczyk Memorial Hospital, Bydgoszcz, Poland, from November 2015 to June 2017. The participants were enrolled before surgery and systemic adjuvant treatment. Data acquisition and analysis were performed in compliance with protocols approved by the local bioethical committee and in accordance with the principles embodied in the Declaration of Helsinki (permission no. KB 547/2015). Written informed consent was obtained from each patient before enrolment and blood sampling.

### 2.2. Study Design

All the patients were preoperatively examined by oncologists in the hospital. The pre-treatment data, including reproductive history, menopausal status, menopausal hormone therapy, comorbidities, history of breast and other cancers, premedication, treatment regimen, intra-hospital outcome at discharge and mortality, duration of the hospital stay, and body mass index, were collected. A menopause was defined as the amenorrhea for 12 months. The body mass index (BMI) (weight in kg/height in m^2^) was calculated from the patient’s height and weight as measured at the initial patient visit, when the individual was wearing no shoes and few clothes. The tumour size, lymph node status and tumour stage were determined; immunohistochemical (IHC) analysis of the oestrogen receptor (ER), progesterone receptor (PR), human epidermal growth factor receptor 2 (HER2) and Ki67 proliferative marker was performed; and the tumours were graded according to the Nottingham Histologic Score system (also termed the Elston–Ellis modification of the Scarff–Bloom–Richardson grading system). The tumour size was defined as the maximum diameter of the sample. The TNM staging of the disease at initial diagnosis was confirmed according to the American Joint Committee on Cancer (AJCC), 7th edition. The intrinsic subtypes of breast cancer were divided into luminal A, luminal B HER2-positive, luminal B HER2-negative, non-luminal HER2-positive and triple-negative. The histological grading system takes into account three components—glandular/tubular differentiation, nuclear pleomorphisms and mitotic counts—each scored from 1 to 3 points; the scores are then summed to a final total score ranging from 3 to 9. There are 3 grades, including low (grade 1), moderate (grade 2) and high (grade 3). Data related to adjuvant treatment, including the administration of adjuvant chemotherapy, immunotherapy, hormonal therapy and radiation therapy, were also collected.

### 2.3. Inclusion and Exclusion Criteria

The eligibility criteria for the patients included (1) a histologically confirmed core needle biopsy stage IA–IIB invasive, primary, unilateral breast cancer; (2) complete clinical records and follow-up data; (3) adequate haematological, liver and renal function; (4) all the peripheral blood samples having been collected within 24 h before surgery. The exclusion criteria encompassed (a) bilateral breast cancer, (b) a carcinoma in situ, (c) neoadjuvant chemotherapy, neoadjuvant hormone therapy or neoadjuvant immunotherapy (d) a history of any cancer, (e) distant metastases, (f) chronic inflammatory diseases or autoimmune disease, (g) morbid obesity (BMI over 40 kg/m^2^), (h) diabetes mellitus type 2 and (i) psychiatric illness.

### 2.4. Follow-Up Details

Cumulative survival was expressed through Kaplan–Meier graphs. The follow-up times were calculated from the date of the initial visit until the earliest event of interest, i.e., disease spread or death, whichever occurred first. Up-to-date survival data were collected at the end of February 2021 and are expressed in months. Relapse was confirmed as signs of metastatic disease or local recurrence as detected by PET/CT or death (excluding deaths unrelated to the disease).

### 2.5. Methods

#### 2.5.1. Blood Sampling and Laboratory Tests

Venous blood was collected from all the subjects at the time of enrolment into tubes containing 1.8 mg of ethylenediaminetetraacetic acid (EDTA). The samples were processed according to standard protocols for blood samples. All the clinicopathological data were obtained as part of routine care. The blood samples were centrifuged at 3000× *g* for 15 min and then stored at −80 °C until analysis. There was no variation in the average storage time between the samples. 

SDF-1α was measured in the patients’ frozen EDTA plasma using commercially available ELISA (enzyme-linked immunosorbent assay) kits (SDF-1α, Cloud-Colne Corp., Houston, TX, USA), whereas VEGF-A, sVEGFR1 and sVEGF2 were measured as previously described [14]. The reaction mixtures were added to 96-well plates.

#### 2.5.2. Cut-Off Values for Analysed Variables

The subjects were distinguished as having low or high values, dichotomised using a cut-off for SDF-1α of 42 pg/dL, based on the median value for the whole study population. The respective cut-off for circulating EPCs was 9.68 cell/μL, and the cut-offs for VEGF-A, sVEGFR1, sVEGFR2 and the sVEGFR2/sVEGFR1 ratio were 64.11 pg/mL, 29.86 pg/mL, 9680.08 pg/mL and 367.04, respectively.

#### 2.5.3. Immunophenotyping

The number of circulating EPCs was determined using a method and recommendations provided by Mancuso et al. and our previous study [15,16]. Pre-treatment blood samples collected with EDTA were analysed on the FACSCaliburTM (BD Bioscience, San Jose, CA, USA) for four-color immunophenotyping. The acquisition was set at 100,000 events (leucocytes) per tube to enumerate the circulating EPCs. We defined circulating EPCs as cells negative for the hematopoietic marker CD45 (using a PerCP-Cy5.5-conjugated anti-CD45 antibody) and positive for the endothelial markers CD31 (fluorescein isothiocyanate (FITC)-conjugated anti-CD31), CD34 (APC-conjugated anti-CD34 antibody) and CD133 (phycoerythrin (PE)-conjugated anti-CD133 antibody; Miltenyi Biotec, Bergisch Glabdach, Germany), a progenitor marker. The antibodies against CD45, CD31 and CD34 were purchased from BD Biosciences, Pharmingen, San Diego, USA. Circulating EPCs were thus defined according to the following immunophenotype: CD45−CD34+CD133+CD31+. The laboratory procedure is described in our former paper [16].

#### 2.5.4. Immunohistochemical Detection of Hormone Receptors, Ki67 and HER2

The hormone receptors ER, PR, HER2 and Ki67 were assessed by immunohistochemistry according to the manufacturer’s indications. According to the staining intensity, the pathologist defined the HER2 status as positive (IHC score = 3+), negative (IHC score = 0 or 1+) or equivocal (inconclusive IHC score = 2+). Oestrogen receptor expression (ER) and progesterone receptor expression (PR) were expressed as positive or negative. The ER and PR variables were then categorised into combined hormone receptor expression (ER+PR+ or ER+PR-/ER-PR+/ER-PR-), as previously published [16].

### 2.6. Statistical Methods

All statistical analyses were conducted using Statistica v. 13.1 (StatStoft^®^, Cracow, Poland). The Shapiro–Wilk test was used to test the normality, and the Mann–Whitney and ANOVA Kruskal–Walis tests were used to compare subgroups. The non-normally distributed variables were described by medians and interquartile ranges (IQR), while the clinicopathologic categorical variables were presented as numbers and percentages (%). Survival curves were plotted using the Kaplan–Meier method and compared between groups using the log-rank test. Univariate and multivariate Cox proportional hazards regression analyses were performed to calculate the hazard ratios and 95% CIs for SDF-1α, angiogenic variables, circulating EPCs and survival. Finally, to evaluate the markers’ ability to predict disease recurrence, analysis of the receiver operating characteristics curve (ROC) by the area under the curve (AUC) was performed according to logistic regression. The optimal cut-off points were determined according to the Youden criteria. Differences with *p*-values less than 0.05 were considered to be statistically significant.

## 3. Results

### 3.1. Patient-Specific Data

We performed an explorative, prospective, single-centre cohort study. Our cohort was 84 patients with IBrC with a median age of 55 years (IQR, 49–59 years). The study was conducted between November 2015 and June 2017. The median follow-up duration for disease-free survival was 58 months (IQR, 52–62 months). During follow-up, 15 events occurred, including 8 deaths and 7 disease recurrences. The patient characteristics can be found in Table 1. Most of the women (46%) had normal (≤24.9 kg/m^2^) body mass indices (BMIs), 36% were overweight (BMI, 25–29.9 kg/m^2^), and 18% were obese (BMI ≥ 30 kg/m^2^). Tumour size was determined according to pathology reports—it was <2 cm in 55 (65%) patients and 2–5 cm in 29 (35%). The median tumour diameter was 1.68 cm (IQR: 1.2–2.1 cm). Regional lymph nodes were involved in one quarter of the patients (positive vs. negative, 24% vs. 76%, respectively). There were 40 (48%) patients with stage IA (T1N0M0—T1 tumours without nodal micrometastases) and 38 (45%) cases with stage IIA (T1N1M0—T1 tumours with nodal micrometastases or T2N0M0—T2 without nodal micrometastases), while six cases (7%) had stage IIB, which was defined by T2N1M0—T2 with nodal micrometastases. Breast-conserving surgery was performed in 82% of the women, and 18% underwent mastectomy. Adjuvant treatments included radiotherapy, chemotherapy, hormone therapy and immunotherapy, which were administered to 83%, 55%, 88% and 8% of the patients, respectively.

The subjects with low (<42 pg/dL) and high SDF-1α (>42 pg/dL) concentrations constituted 48% and 52% of the study group, respectively (Table 2). Patients under 55 years and premenopausal had lower SDF-1α concentrations (24 subjects and 21 cases, respectively) than those 55 years or over and postmenopausal (28 and 38 patients, respectively); the differences were statistically significant (*p* = 0.0303 and 0.0001, respectively). Additionally, in Figures S1 and S2 a positive correlation between a pre-treatment concentration of SDF-1α with patients’ age (r = 0.2364; *p* = 0.0304) and a positive correlation between a pre-treatment concentration of SDF-1α with patients’ menopausal status (r = 0.4156; *p* = 0.0001) were presented.

### 3.2. Analysis of SDF-1α and Selected Vasculo-Angiogenic Factors as Prognostic Markers Using Univariate and Multivariate Cox Proportional Hazards Regression Models

The multivariate Cox regression model adjusted for prognostic factors including BMI, age at the time of diagnosis, staging system, intrinsic type, histological type, nodal involvement and tumour diameter showed a decrease in the risk of disease relapse with an increase in the concentration of SDF-1α and the number of circulating EPCs (HR = 5.1263; 95% CI, 1.0774–24.3915; *p* = 0.0400; HR = 7.9152; 95% CI, 1.2207- 51.3243; *p* = 0.0301, respectively) for DFS. Thus, subjects with an SDF-1α concentration higher than 42 pg/mL appear to have a 5.13-times-lower risk of disease recurrence; also, patients with numbers of circulating EPCs higher than 9.68 cell/μL have a 7.91-times-lower risk of disease relapse. With respect to selected angiogenic parameters according to the multivariate Cox regression model adjusted for age, BMI and cancer-related prognostic factors showed that patients with an sVEGFR1 concentration higher than 29.86 pg/mL present 4-times better survival outcomes (HR = 4.0111; 95% CI, 1.6381–166.2917; *p* = 0.0271). However, the concentration of VEGF-A and the sVEGFR2/sVEGFR1 ratio indicated opposite associations for DFS (HR = 0.0956 95% CI, 0.0122–0.7485; *p* = 0.0254; HR = 0.0184; 95% CI, 0.0011–0.3062; *p* = 0.0053, respectively).

Also, the univariate Cox regression model showed a decrease in the risk of disease relapse with an increase in the SDF-1α concentration (HR = 3.2645; 95% CI, 1.0375–10.2717; *p* = 0.0431); moreover, a higher number of circulating EPCs was related to better DFS (HR = 3.2619; 95% CI, 1.0341–10.2894; *p* = 0.04367). According to our data, subjects with an SDF-1α concentration higher than 42 pg/mL appear to have a 3.26-times-lower risk of disease recurrence, and patients with numbers of circulating EPCs higher than 9.68 cell/μL have a 3.26-times-lower risk of disease relapse (Table 3).

### 3.3. Assessment of the Analysed Parameters’ Ability to Predict Disease Recurrence, According to the Receiver Operating Characteristics Curve (ROC)

**Figure 1:**
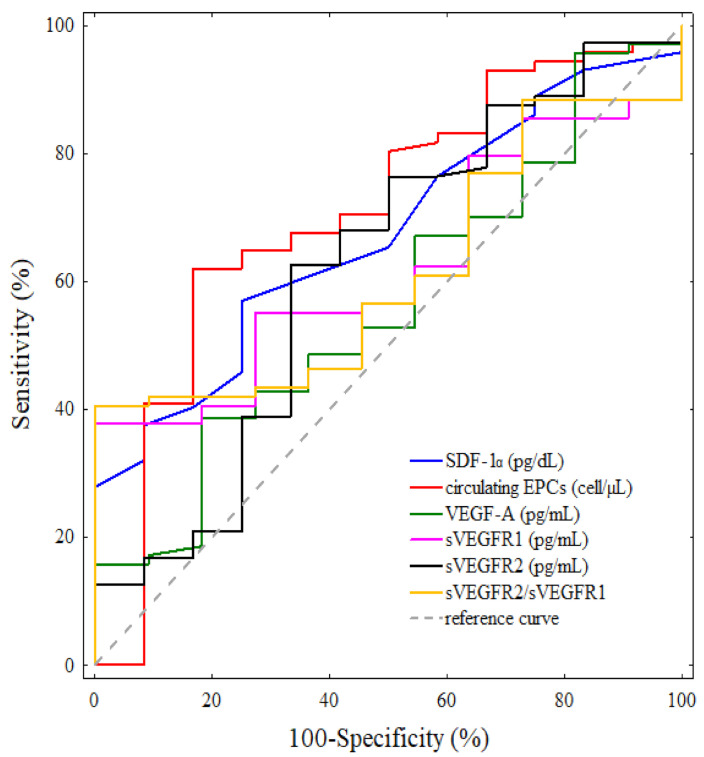
Receiver operating characteristic (ROC) curves for single biomarker detection in predicting disease-free survival in IBrC patients. SDF-1α- AUC = 0.674; *p* = 0.0162, circulating EPCs- AUC = 0.708; *p* = 0.0131, VEGF-A- AUC = 0.568; *p* = 0.4410, sVEGFR1- AUC = 0.622; *p* = 0.0906, sVEGFR2- AUC = 0.620; *p* = 0.1990, sVEGFR2/sVEGFR1- AUC = 0.613; *p* = 0.1267.

We analysed the association between the pre-treatment concentrations of SDF-1α, VEGF-A, sVEGFR1 and sVEGFR2 and the sVEGFR2/sVEGFR1 ratio as well as the number of circulating EPCs with respect to disease relapse during the 58-month follow-up of the IBrC patients (Figure 1). ROC curves for separate laboratory determinants were designed, and the areas under the curve (AUC) with 95% CIs were determined (AUC, 95% CI threshold with sensitivity and specificity). We appraised the ROC curves in order to estimate the diagnostic accuracies of the investigated variables for the prediction of disease recurrence. An area under the ROC curve (AUC^ROC^) ≥ 0.5 (*p* ≤ 0.05) was found only for SDF-1α and circulating EPCs. Although the AUC^ROCs^ for VEGF-A, sVEGFR1, sVEGFR2 and the sVEGFR2/sVEGFR1 ratio were above 0.5, the *p*-values were >0.05. The Youden index cut-off values for SDF-1α and circulating EPCs maximising the sum of the sensitivity and specificity were determined. For SDF-1α, the AUC^ROC^ value was 0.674 (*p* = 0.0162). The Youden index cut-off value for the plasma SDF-1α concentration best discriminating between patients at high and low risk of relapse was determined to be 42 pg/dL, resulting in a sensitivity of 57% and specificity of 75%. The SDF-1α area under the ROC curve was lower than the area for circulating EPCs (AUC^ROC^ = 0.708, *p* = 0.0131). According to the receiver operating characteristic curve for circulating EPCs, the appropriate Youden index cut-off value was determined to be 8.53 cells/µL, providing a 62% sensitivity and 83% specificity (Figure 1).

**Figure 2:**
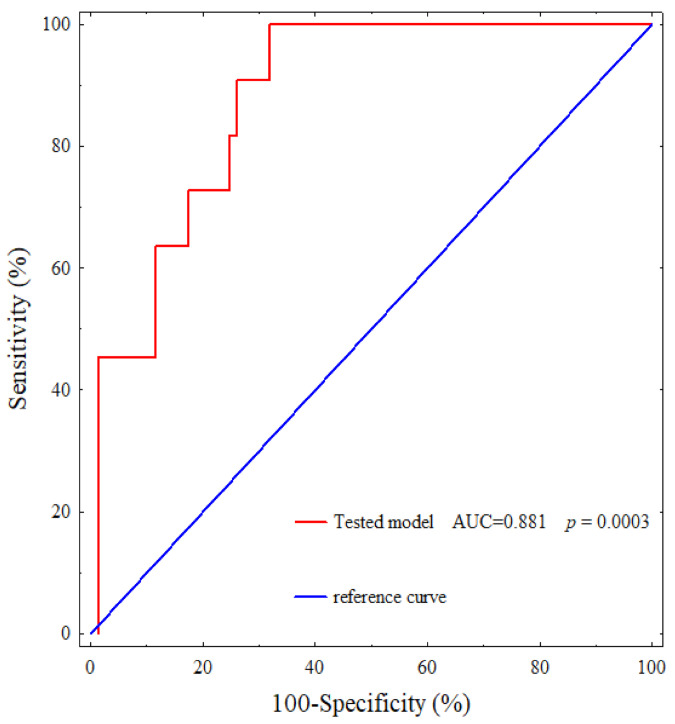
Receiver operating characteristic (ROC) curve for the tested model in predicting disease-free survival in IBrC patients.

As well as the ROC curves for the separate laboratory parameters, we generated a ROC curve for the tested model consisting of the SDF-1α, VEGF-A, sVEGFR1, sVEGFR2 concentrations and the number of circulating EPCs as well as the sVEGFR2/sVEGFR1 ratio. For all these parameters, the area under the curve was 0.881 (*p* = 0.0003) (Figure 2). Thus, this combination of parameters involved in vasculogenesis, angiogenesis and chemotaxis demonstrates a deeper molecular link between these processes but also great prognostic usefulness.

### 3.4. Predictive Value of Pre-Treatment Values of SDF-1α (A), Circulating EPCs (B), VEGF-A (C), sVEGFR1 (D), sVEGFR2 (E) and sVEGFR2/sVEGFR1 Ratio (F) for Survival

**Figure 3:**
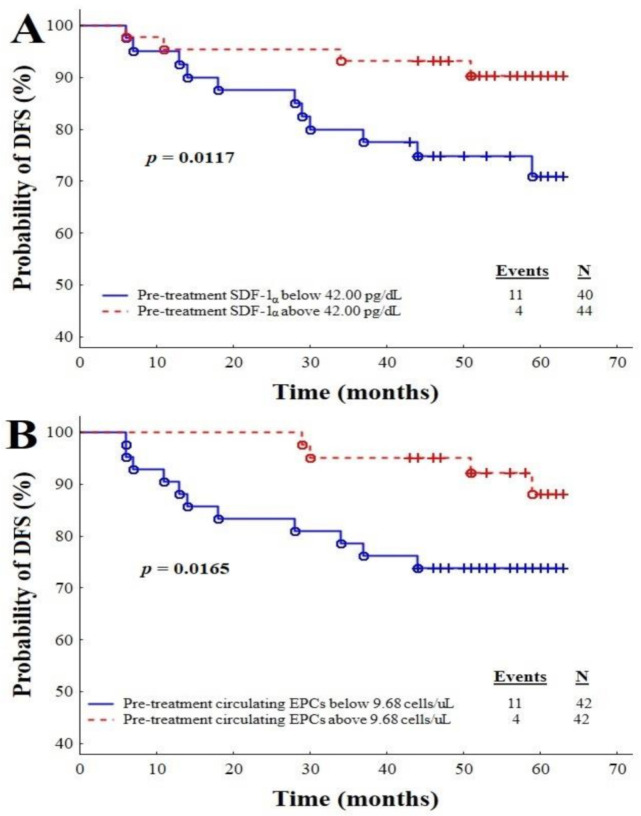
Plots A and B: Kaplan–Meier survival curves showing pre-treatment plasma SDF-1α (**A**) and the number of circulating EPCs (**B**) in IBrC patients, divided according to cut-offs: for SDF-1α, <42 pg/dL and >42 pg/dL; circulating EPCs, <9.68 cells/µL and > 9.68 cells/µL.

Based on the ROC analysis for the single analysed variable as well as the univariate and multivariate Cox regression models, the additional statistical analysis was performed in order to support the prognostic utility of SDF-1α and circulating EPCs in disease relapse. The Kaplan–Meier curves for DFS (Figure 3A,B) show the effects of the pre-treatment concentrations of SDF-1α and the number of circulating EPCs on the survival of IBrC patients, during the 58-month follow-up. During the follow-up period, 15 of the 84 patients (recurrence rate, 17.85%) had relapse, and 8 died due to systemic metastatic disease. A concentration of SDF-1α lower than 42 pg/dL was associated with shorter DFS in the IBrC patients (*p* = 0.0117) (Table S1). Forty out of the 84 cases had concentrations of SDF-1α lower than 42 pg/dL; the recurrence rate for those subjects was 27.5% versus 9.09% for those with higher levels. Having a number of circulating EPCs lower than 9.68 cells/µL was also linked with shorter DFS (*p* = 0.0165); the recurrence rate was 26.19% for the patients with circulating EPC counts lower than 9.68 cells/µL, compared to 9.52% for those with its higher counts.

**Figure 4:**
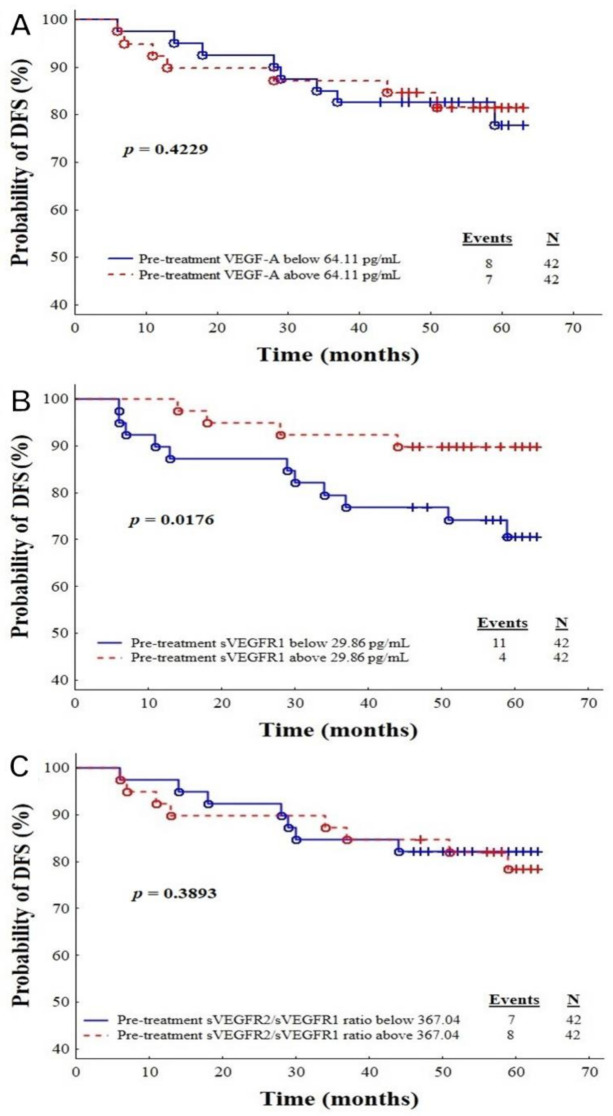
Plots C-E: Kaplan–Meier survival curves showing pre-treatment plasma VEGF-A (**A**), plasma sVEGFR1 (**B**) and sVEGFR2/sVEGFR1 ratio (**C**) in IBrC patients, divided according to cut-offs: for VEGF-A, <64.11 and >64.11 pg/mL; sVEGFR1, <29.86 and >29.86 pg/mL and sVEGFR2/sVEGFR1 ratios, <367.04 and >367.04.

Additionally, based on the multivariate Cox regression model, we have provided Kaplan–Meier curves for the VEGF-A (Figure 4A), sVEGFR1 (Figure 4B) and sVEGFR2/sVEGFR1 ratio (Figure 4C). A significant difference with respect to the concentration of sVEGFR1 was noted (Figure 4). The recurrence of the disease in the group of patients with sVEGFR1 concentrations higher than 29.86 pg/mL occurred in four out of 42 (9.52%), but in the subgroup with sVEGFR1 levels lower than 29.86 pg/mL, 11 out of 42 (26.19%) had recurrence (*p* = 0.0176). 

### 3.5. Survival Time Analysis with Respect to the Combinations of SDF-1α with Circulating EPCs (5A), SDF-1α with sVEGFR1 (5B), SDF-1α with VEGF-A (6A), SDF-1α with sVEGFR2 (6B), and SDF-1α with the sVEGFR2/sVEGFR1 Ratio (6C)

**Figure 5:**
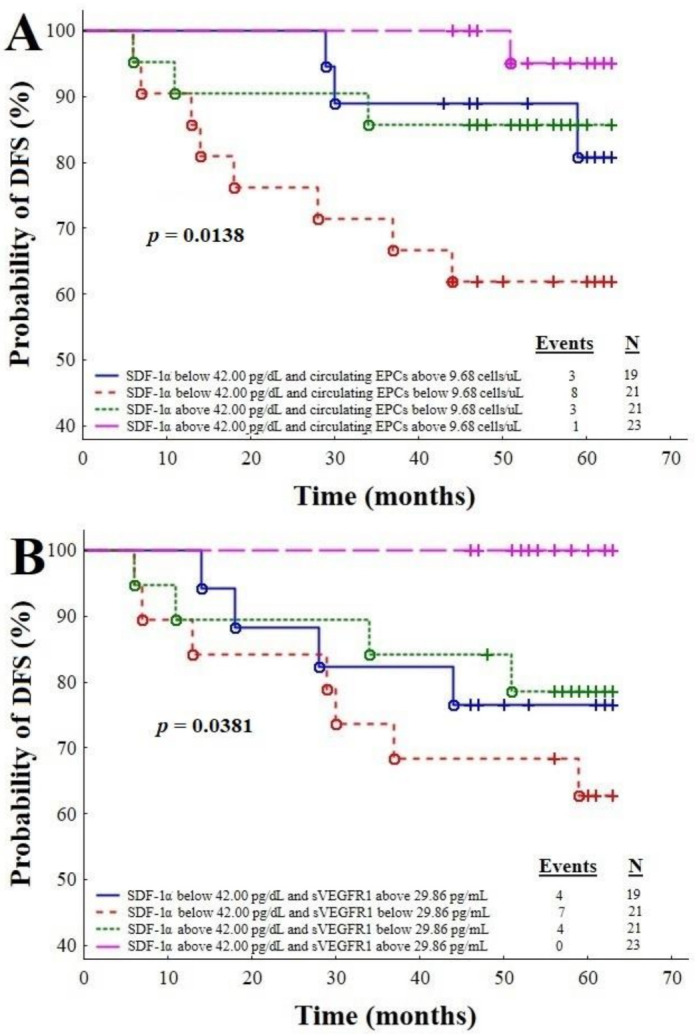
Plots A and B: Combinations of the SDF-1α level with the number of circulating endothelial progenitor cells (**A**), and the SDF-1α level with sVEGFR1 (**B**) in predicting disease relapse.

**Figure 6:**
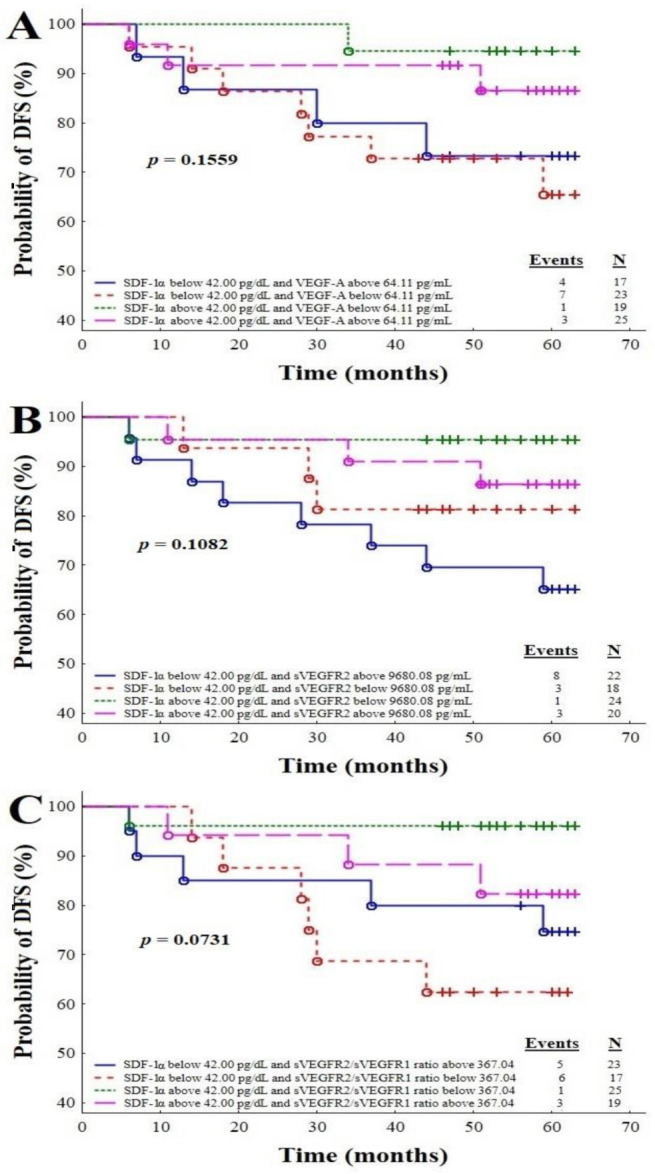
Plots A, B and C: Combinations of the SDF-1α level and VEGF-A (**A**), the SDF-1α level and sVEGFR2 (**B**), and the SDF-1α level and sVEGFR2/ sVEGFR1 ratio (**C**) for the prediction of disease relapse.

The prognostic value of the combinations of SDF-1α with circulating EPCs, SDF-1α with sVEGFR1, SDF-1α with VEGF-A, SDF-1α with sVEGFR2, and SDF-1α with the sVEGFR2/sVEGFR1 ratio in IBrC patients was assessed (Figure 5 and Figure 6). This analysis was based on results of the ROC curve for the tested model consisting of the SDF-1α, VEGF-A, sVEGFR1, and sVEGFR2 concentrations and the number of circulating EPCs as well as the sVEGFR2/sVEGFR1 ratio. This combination of parameters involved in vasculogenesis, angiogenesis and chemotaxis demonstrates a great prognostic usefulness (AUC^ROC^ = 0.881). The combination of SDF-1α with the number of circulating EPCs showed interesting results: Figure 5A demonstrates a significantly better IBrC specific survival for subjects with higher SDF-1α (>42 pg/mL) and a higher number of circulating EPCs than 9.68 cells/µL with respect to cases with SDF-1α level < 42 pg/mL and a number of circulating EPCs lower than 9.68 cells/µL (*p* = 0.0138). Recurrence of the disease in the group of higher SDF-1α than 42 pg/mL and a higher number of circulating EPCs than 9.68 cells/µL occurred in one out of 23 (4.35%) cases, but, in the subgroup SDF-1α level < 42 pg/mL and a number of circulating EPCs lower than 9.68 cells/µL, eight out of 21 (38.09%) cases. A similar spectacular effect was obtained with respect to the combination patients with SDF-1α > 42 pg/dL and sVEGFR1 > 29.96 pg/mL, since those subjects had longer DFS than those with SDF-1α < 42 pg/dL and sVEGFR1 < 29.96 pg/mL (*p* = 0.0381, Figure 5).

Figure 6 presents the Kaplan–Meier DFS curves for the IBrC patients analysed on the basis of the SDF-1α and VEGF-A (Figure 6A) combination and the SDF-1α and sVEGFR2 (Figure 6B) combination, as well as the combination of SDF-1α and a sVEGFR2/sVEGFR1 ratio (Figure 6C). According to those analyses, combinations of the above-mentioned parameters did not predict disease relapse (*p* = 0.1559, 0.1082, 0.0731, respectively). 

## 4. Discussion

Despite the serious advances in the early diagnosis and therapy of breast cancer, metastasis remains the leading cause of mortality for this disease. Genomic changes in the tumour cells and microenvironment, as well as the biological properties of the host and target tissue, drive cancer transformation and metastasis. These actions depend on various signals such as growth factors, cytokines, chemokines and extracellular matrix modifications [17]. The interaction of SDF-1α and CXCR4 plays an important role in different aspects of tumour progression—including cell proliferation, survival and chemotaxis [7]—as well as establishing metastasis in particular tissues. Furthermore, the SDF-1α/CXCR4 axis participates in tumour neoangiogenesis. SDF-1α is released by cancer-associated fibroblasts and recruits endothelial progenitor cells and induces the expression of VEGF, which leads to the development of the tumour blood vessel network. There is also a mutual interaction between the SDF-1α/CXCR4 axis and VEGF. Both SDF-1α and VEGF-A play important roles in the formation of new tumour vasculature. The expression of VEGF on endothelial cells is enhanced by SDF-1α, and VEGF, in turn, induces CXCR4 expression on those cells, thus triggering neoangiogenesis [18].

### 4.1. Pre-Treatment Values of SDF-1α as a Prognostic Indicator

We assessed whether the plasma level of SDF-1α could act as a biomarker for the risk of disease relapse or progression in invasive breast cancer patients. In our study, patients younger than 55 years and premenopausal had lower serum levels of SDF-1α, so those factors might be negative indicators. We further confirmed that plasma levels of SDF-1α lower than 42 pg/dL were associated with a poorer prognosis and shorter DFS. Based on the ROC curve, a cut-off SDF-1α concentration of 42 pg/dL best discriminates between patients at high and low risk of relapse, with a sensitivity of 57% and specificity of 75%. Also, the univariate and multivariate Cox regression models showed that an SDF-1α concentration higher than 42 pg/mL is associated with a 3.26-times-lower and a 5.13–times-lower risk of disease recurrence, respectively. Our findings are in line with Hassan et al. The authors found no significant association between SDF-1α and tumour size, grade, stage, hormone receptor status or HER2 overexpression, nor did we find such associations. Similar to our study, Hassan et al. reported that low plasma SDF-1 levels were predictive of distant metastasis and also an independent prognostic marker for poorer breast cancer–specific survival. However, it is worth mentioning that the study group in Hassan et al.’s study included patients with more advanced disease as well as those who received neoadjuvant hormone treatment or chemotherapy, in contrast to our study, in which neoadjuvant treatment was an exclusion criterion. The authors of the mentioned study stated that a clinically relevant step in breast cancer metastasis occurs at tumour extravasation; there is a differential concentration gradient due to lower SDF-1α levels in the circulation than in the metastatic organ site [19]. It was already demonstrated that the SDF-1α receptor, CXCR4, participates in the homing of breast cancer cells, mainly in the bones and liver [20,21]. CXCR4 is overexpressed in breast cancer cells circulating in the blood, and an association between the plasma level of CXCR4 and circulating tumour cells was confirmed. Furthermore, in a study by Kong et al., SDF-1α overexpression in the breast cancer MCF-7 cell line promotes the proliferation, invasion and migration of tumour cells. Epithelial–mesenchymal transition (EMT) and the EMT-induced acquisition of the cancer stem cell (CSC)-like phenotype, which are crucial for cancer cell invasion and metastasis, are triggered by the overexpression of SDF-1α through the NF-κB pathway [22]. However, there is no correlation between SDF-1α expression in tumour cells and the number of circulating tumour cells in the blood, nor do plasma and tissue SDF-1α levels correlate. This implies that the main origin of plasma SDF-1α is not the breast tumour [23]. The sources of plasma SDF-1α are multiple, including cells such as cancer-associated fibroblasts or myofibroblasts, lymphocytes, endothelial cells and bone marrow [19,24]. The higher SDF-1α levels in the blood of breast cancer patients might attach to cancer cells, preventing their homing to metastatic sites and thus improving the prognosis [19]. The SDF-1α/CXCR4 axis plays an important role in the mobilisation and homing of circulating tumour cells [23]. A large meta-analysis by Samarendra et al., which included 5807 patients with different cancer types, including breast cancer, revealed that SDF-1α expression may become a cancer biomarker and indicate patients’ prognoses. High SDF-1α expression was associated with worse prognoses in oesophagogastric, pancreatic and lung cancer. By contrast, increased SDF-1α expression in breast cancer was found to be a marker of better overall survival, which is consistent with our study. However, the cause of these divergent results for different types of cancer is unclear. In explaining this discrepancy, the authors considered the different sources of SDF-1α in the included studies; the primary source of SDF-1α tended to be the tumour cells. It is possible that stromal and tumour cell SDF-1α production have different roles in cancer progression [25]. Furthermore, SDF-1α plays an important role in the local invasion of the tumour, while the loss of its expression leads to tumour cells migration to organs expressing high levels of SDF-1α (mainly the liver, bones and lungs). Hence, breast cancer metastasis is associated with the downregulation of SDF-1α in cancer cells, while in pancreatic, oesophagogastric and lung tumours, high SDF-1α expression leads to local invasion and is thus associated with poorer outcomes, as these cancers mainly cause mortality via local invasion [25].

### 4.2. Pre-Treatment Values of SDF-1α with Respect to Clinicopathological Features

It has already been established that SDF-1α and its receptor CXCR4 are expressed in breast cancer cells and that their activation contributes to angiogenesis and the successive steps of metastasis [26]. CXCR4 supports the formation of metastases in organs where SDF-1α is released in large amounts, such as the bones, liver and lungs. Circulating tumour cells leave the blood vessels as a result of the interplay between SDF-1α and CXCR4, which leads to metastatic tumour formation [27]. The presence of SDF-1α may boost the infiltration and mobility of breast cancer cells, and its expression is correlated with lymph node metastases and long-term survival in affected individuals. As SDF-1α may lead to the invasion and spread of tumour cells, its expression is correlated with clinical outcomes: there were significantly higher levels of SDF-1α RNA transcript in tumour cells in patients with local recurrence and in those who died from breast cancer. Furthermore, in patients with metastases in lymph nodes, there were higher levels of SDF-1α transcript in tumour cells [5]. Interestingly, Wu et al. noted that different intrinsic subtypes of breast cancer differ with the expression of SDF-1α. In HER2-positive and triple-negative breast cancer tissues, expression of SDF-1α was significantly higher than in luminal A and luminal B subtypes, but there is no difference in SDF-1α expression between luminal A and luminal B subtypes or between HER2-positive and triple-negative breast cancer cells [28]. However, this observation is inconsistent with our study. Thus, according to these discrepancies, further studies are needed in this regard.

### 4.3. Pre-Treatment Number of Circulating Endothelial Cells as a Prognostic Indicator

Based on unadjusted univariate and adjusted multivariate regression models and single-biomarker ROC and Kaplan–Meier survival curves, we found that apart from low SDF-1α (<42 pg/dL) levels, a low number of circulating EPCs (<9.68 cells/µL) may also serve as a single biomarker for shorter DFS in breast cancer patients. The univariate and multivariate Cox regression models demonstrated that the numbers of circulating EPCs higher than 9.68 cell/μL had a 3.26-times-lower risk and a 7.91–times-lower risk of disease relapse, respectively. Interestingly, patients with low SDF-1α levels combined with low numbers of circulating EPCs had the shortest DFS in our study. The role of circulating EPCs in the cancer’s development is not entirely understood, but carcinoma patients are characterised by higher numbers of circulating EPCs than healthy individuals [29]. Several studies have shown elevated circulating EPCs to correlate with more advanced disease stages and worse prognoses in certain haematological and solid cancers [30]. Dome et al. found that in non-small-cell lung carcinoma patients, higher circulating EPC numbers before treatment correlated with poorer overall survival. Furthermore, in the subgroup of responders to treatment, the post-treatment EPC numbers in the peripheral blood were significantly lower than in the nonresponding patients [31]. In studies by Richter-Ehrenstein et al. and Naik et al., higher preoperative numbers of EPCs in breast cancer patients were associated with larger tumour sizes or higher disease stages; the number of EPCs immediately dropped after the resection of the tumour or initiation of chemotherapy [32,33]. Furthermore, Jain et al. observed an increase in the median EPC number prior to clinical relapse or the progression of the disease; in responding patients, the median EPC number decreased [34]. However, not all cancer studies show higher numbers of circulating EPCs to be associated with worse prognoses. In a study by Rhone et al., treatment conducted as in previous studies significantly reduced the number of EPCs/µL in the general IBrC cohort. Interestingly, a low pre-treatment count of circulating EPCs was associated with a higher risk of breast cancer relapse, consistent with our results [16]. Botelho et al. claimed that breast tumour cells recruit EPCs in a very specific manner; they demonstrated that the tumours engage EPCs during transformation and that endothelial progenitors are no longer needed when the tumours reach the plateau phase of growth [35]. This theory is in line with our findings. Additionally, the combination of the number of circulating EPCs with the concentration of SDF-1α has prominent diagnostic value. Postnatally, SDF-1α plays an important role in stimulating the recruitment of circulating EPCs from the bone marrow through a CXCR4-receptor-dependent mechanism [36]. Presumably, an increase in the SDF-1α concentration with a simultaneous increase in the number of EPCs indicates a better attraction and control of cancer cells and superior stability of the vascular network. In summary, the number of circulating EPCs and the SDF-1α concentration may serve as markers of the response to treatment and prognosis in breast cancer patients.

### 4.4. Pre-Treatment Concentrations of Selected Angiogenic Parameters as Prognostic Indicators

The binding of membrane-bound receptors with VEGF increases the permeability, proliferation and migration of vascular endothelial cells. The activation of VEGFR2 is responsible for mediating the major actions of VEGF, including growth and increased vascular permeability. The higher affinity of VEGFR1 for VEGF blocks VEGFR2 activation. However, the soluble forms of VEGF receptors type 1 and 2 are believed to be natural suppressors of VEGF activity; they control the intensity of neoangiogenesis [37,38]. 

Our study has important theoretical and clinical implications. Firstly, the presented model—using the plasma concentrations of SDF-1α, VEGF-A, sVEGFR1 and sVEGFR2 and the sVEGFR2/sVEGFR1 ratio, as well as the number of circulating EPCs, for predicting disease-free survival in breast cancer patients—may help to identify patients with poorer prognoses, who would benefit from more intense treatment. The combination of the parameters involved in vasculogenesis, angiogenesis and chemotaxis demonstrates that there are profound molecular interactions between these processes. As well as their possible use in the diagnosis and prognosis of breast cancer recurrence, it has been shown that blocking the CXCR4/SDF-1α axis inhibits the growth of breast cancer in vivo and in vitro [27]. Interestingly, according to the multivariate regression model, a higher concentration of VEGF-A and a higher sVEGFR2/sVEGFR1 ratio are associated with poorer prognosis. The up-regulation of VEGF-A is linked with neovascularisation and the pro-metastatic phenotype of cancer cells and shorter survival rate [37,38].

Furthermore, the Kaplan–Meier survival analysis showed that sVEGFR1 as a single indicator as well as combined with the SDF-1α is able to predict future outcomes. Nevertheless, the recurrence rate for IBrC cases with SDF-1α < 42 pg/mL and sVEGFR1 < 29.86 pg/mL was 33.33% versus 0% for their SDF-1α > 42 pg/mL and sVEGFR1 > 29.86 pg/mL counterparts. Our study reinforces the concept that the plasma levels of SDF-1α indicate the ability to bind circulating cancer cells expressing CXCR4 and, thus, prevent their homing to possible metastatic sites. Based on this hypothesis, the idea of inhibiting the CXCR4/SDF-1α axis has mainly concentrated on inhibiting the receptor CXCR4. Furthermore, other extracellular proteins involved in the CXCR4 pathway (such as HER2) show some therapeutic potential for the inhibition of breast cancer metastases [39]. However, more research is needed through breast cancer clinical trials to determine the efficacy of CXCR4/SDF-1α antagonists alone or in combination with other chemotherapeutic agents.

### 4.5. Strengths and Limitations of the Study

Our study has several strengths. Firstly, all the blood samples were collected prospectively, one day before surgery, in fasting patients, minimizing the fluctuations that could have occurred if the samples were collected at different time points. Furthermore, this study used patients at an early stage of the (stages IA-IIB) disease who were relatively homogenous, and excluded patients with advanced cancers or with co-existing diseases that could have affected the test results, thereby minimizing the risk of bias. However, there are also some limitations to this study. Firstly, the median follow-up was only 4.83 years (the median follow-up duration for disease-free survival was 58 months (IQR, 52–62 months); a longer follow-up would be necessary to investigate distant recurrences occurring beyond this point. Finally, the sample size was relatively small, so our findings need to be confirmed in a larger population. Due to the fact that our study had an observational, prospective nature and was performed in a daily clinical practice setting, the sample size was dependent on obtaining patients’ consent for participation and meeting by patients very restricted inclusion criteria.

## 5. Conclusions

In conclusion, even though this study enrolled a relatively small population of IBrC patients and the findings should be interpreted with caution, our results raise several important points: (1) IBrC patients with lower pre-treatment plasma levels of SDF-1α (<42 pg/dL) and lower numbers of circulating EPCs (<9.68 cells/µL) have worse prognoses. (2) SDF1-α in combination with circulating EPCs and SDF1-α with sVEGFR1 has strong prognostic value for predicting breast cancer recurrence and may be helpful in identifying patients at increased risk of disease relapse, who require more aggressive approaches to therapy. (3) A combination of parameters involved in vasculogenesis, angiogenesis and chemotaxis demonstrates great prognostic usefulness. (4) Finally, the SDF1-α/CXCR4 axis is involved in progression and metastasis; however, its role requires further investigation before new approaches to treatment can be routinely implemented in the clinic.

## Figures and Tables

**Table 1:** Pre-treatment demographic and clinical characteristics of the study population.

Patient Characteristics	Number of Patients (%)
No of patients: *n* = 84 females	84 (100%)
Age at diagnosis (years)	
<55	40 (48%)
≥55	44 (52%)
BMI kg/m^2^ according to WHO criteria:	
Normal weight (<24.99)	39 (46%)
Overweight (≥25–29.99)	30 (36%)
Obese (30 or more)	15 (18%)
Menopausal status:	
Pre-menopausal	27 (32%)
Post-menopausal	57 (68%)
Parity:	
Nulliparous/1–2 children/≥3 children	6 (7%)/62 (74%)/16 (19%)
Tumour characteristics	
Tumour diameters	
T1 (a, b, c < 2 cm)/ T2 (≥2 cm, but <5 cm)	55 (65%)/29 (35%)
Nodal status (N):	
N0/N1	64 (76%)/20 (24%)
Metastases (M)	None
Stage (TNM classifications)	
IA/IIA/IIB	40 (48%)/38 (45%)/6 (7%)
Histopathological grade:	
Grade 1/ Grade 2/ Grade 3	6 (7%)/67 (80%)/11 (13%)
Histological diagnosis:	
Invasive ductal carcinoma (IDC)	73 (87%)
Invasive lobular carcinoma (ILC)	11 (13%)
Intrinsic subtype	
Luminal A (*ER+/PR+/HER2−/Ki-67 < 20%)*	51 (61%)
Luminal B HER2 *negative* (*ER+/PR+/HER2−/Ki-67 ≥ 20%)*	16 (19%)
Luminal B HER2 *positive* (*ER+/PR+/HER2+/Ki-67- all %)*	7 (8%)
Non-luminal HER2 positive (*ER-/PR-/HER2+/Ki-67- all %)*	2 (2%)
Triple-negative *(ER-/PR-/HER2-/Ki-67- all %)*	8 (10%)
ER status	
positive/negative	74 (88%)/10 (12%)
PR status	
positive/negative	68 (81%)/16 (19%)
HER2 status	
positive/negative	9 (10%)/75 (90%)
Ki67 expression	
<20%/>20%	45 (54%)/39 (46%)
Laterality:	
Left breast/Right breast	45 (54%)/39 (46%)

BMI—body mass index; WHO—World Health Organization; TNM classifications—Tumour diameter (T), Nodal status (N), Metastases (M); ER—oestrogen receptor; PR—progesterone receptor; HER-2—human epidermal growth factor receptor 2; Ki-67—proliferation marker.

**Table 2:** Pre-treatment profiles of the study population based on proportion scores with the cut-off value of 42 pg/mL for the immunoassay regarding SDF-1α.

Attributes	Number of Cases in Each Subgroup	Low SDF-1α (<42 pg/dL)		High SDF-1α (>42 pg/dL)		*p*-Values
		N	%	N	%	
Total number of patients	84	40	48%	44	52%	
Age						**0.0303**
<55 years	40	24	60%	16	40%	
≥55 years	44	16	36%	28	64%	
Menopausal status						**0.0001**
Premenopausal	27	21	78%	6	22%	
Postmenopausal	57	19	33%	38	67%	
Body mass index (kg/m^2^)						0.3237
≤24.9	39	22	56%	17	44%	
25.0–29.9	30	12	40%	18	60%	
30.0–39.9	15	6	40%	9	60%	
Tumour size						0.4057
T1 < 2 cm	55	28	51%	27	49%	
T2 ≥ 2–5 cm	29	12	41%	17	59%	
Nodal status						0.4489
Negative	64	29	45%	35	55%	
Positive	20	11	55%	9	45%	
Stage						0.6770
IA	40	20	50%	20	50%	
IIA+IIB	44	20	45%	24	55%	
ER status						0.2346
Positive	74	37	50%	37	50%	
Negative	10	3	30%	7	70%	
PR status						0.3677
Positive	68	34	50%	34	50%	
Negative	16	6	37.5%	10	62.5%	
HER2 status						0.6139
Positive	9	5	56%	4	44%	
Negative	75	35	47%	40	53%	
Ki67 expression						0.8023
<20%	45	22	49%	23	51%	
>20%	39	18	46%	21	54%	
Histologic grade						0.8751
G1+G2	73	35	48%	38	52%	
G3	11	5	45%	6	55%	
Intrinsic subtype of IBrC						0.5329
Luminal A	51	26	51%	25	49%	
Luminal B HER (−)	16	7	44%	9	56%	
Luminal B HER(+) and Non-luminal HER(+)	9	5	55%	4	45%	
Triple-negative	8	2	25%	6	75%	
Surgery type						0.2216
BCS	69	35	51%	34	49%	
Mastectomy	15	5	33%	10	67%	
Radiotherapy						0.8451
Yes	70	33	47%	37	53%	
No	14	7	50%	7	50%	
Chemotherapy						0.6307
Yes	46	23	50%	23	50%	
No	38	17	45%	21	55%	
Immunotherapy						0.5982
Yes	7	4	57%	3	43%	
No	77	36	47%	41	53%	
Hormone therapy						0.2346
Yes	74	37	50%	37	50%	
No	10	3	30%	7	70%	

SDF-1α—stromal cell-derived factor 1α; ER—oestrogen receptor; PR—progesterone receptor; HER2—human epidermal growth factor receptor 2; Ki67—proliferation marker; G1—well-differentiated (grade 1); G2—moderately differentiated (grade 2); G3—poorly differentiated; BCS—breast-conserving surgery; significant differences are denoted by bold.

**Table 3:** The multivariate and univariate Cox regression models for disease-free survival (R^2^ = 0.9220).

		Multivariate				Univariate		
Variables	HR	95% CI		*p*-Values	HR	95% CI		*p*-Values
SDF-1α								
Low	1.0000	-	-	-	1.0000	-	-	-
High	5.1263	1.0774	24.3915	**0.0400**	3.2645	1.0375	10.2717	**0.0431**
Circulating EPCs								
Low	1.0000	-	-	-	1.0000	-	-	-
High	7.9152	1.2207	51.3243	**0.0301**	3.2619	1.0341	10.2894	**0.04367**
VEGF-A								
Low	1.0000	-	-	-	1.0000	-	-	-
High	0.0956	0.0122	0.7485	**0.0254**	1.1054	0.4007	3.0489	0.8466
sVEGFR1								
Low	1.0000	-	-	-	1.0000	-	-	-
High	4.0111	1.6381	166.2917	**0.0271**	0.9831	0.3539	2.7312	0.9739
sVEGFR2								
Low	1.0000	-	-	-	1.0000	-	-	-
High	1.4369	0.3114	6.6298	0.6422	1.3508	0.4824	3.7826	0.5671
sVEGFR2/sVEGFR1 ratio								
Low	1.0000	-	-	-	1.0000	-	-	-
High	0.0184	0.0011	0.3062	**0.0053**	0.6982	0.2470	1.9733	0.4980

Cox proportional hazards model was used for unadjusted univariate and adjusted multivariate analyses—BMI, age at the time of diagnosis, staging, intrinsic type, histological type, nodal involvement and tumour diameter. HR—hazard ratio; CI—confidence interval; SDF-1α—stromal cell-derived factor 1α; circulating EPCs—circulating endothelial progenitor cells; VEGF-A—vascular endothelial growth factor A; sVEGFR1—soluble form of vascular endothelial growth factor receptor type 1; sVEGFR2—soluble form of vascular endothelial growth factor receptor type 2; significant differences are denoted by bold.

## Data Availability

The data presented in this study are available in this article and supplementary material.

## Supplementary Materials

The following are available online at https://www.mdpi.com/article/10.3390/cancers13081952/s1. Table S1: Correlations between the concentration of SDF-1α and selected clinicopathological attributes in all breast cancer patients, Figure S1: Scatter plot shows a positive correlation between a pre-treatment concentration of SDF-1α with patients’ age (Spearman correlation coefficient, r = 0.2364; *p* = 0.0304), Figure S2: Scatter plot shows a positive correlation between a pre-treatment concentration of SDF-1α with patients’ menopausal status (Spearman correlation coefficient, r = 0.4156; *p* = 0.0001).
