# Supplementary material for: Stromal Cell-Derived Factor 1α (SDF-1α) in Invasive Breast Cancer: Associations with Vasculo-Angiogenic Factors and Prognostic Significance
Source: Cancers (Basel). 2021 Apr 18;13(8):1952. doi: 10.3390/cancers13081952 (PMC8072989; doi:10.3390/cancers13081952)
Supplement: Supplementary file 1 [file cancers-13-01952-s001.zip › cancers-1162356-supplementary.pdf]

Table S1. Correlations between the concentration of SDF-1α and selected clinicopathological attributes in all breast cancer patients

Attributes	SDF-1α R Spearmana	*P*-values
**Age**	0.2364	**0.0304**
**Menopausal status**	0.4156	**0.0001**
**Body mass index (kg/m^2)**	0.1537	0.1629
**Tumour size**	0.0907	0.4118
**Nodal status**	-0.0826	0.4550
**Stage**	0.0455	0.6814
**ER status**	0.1297	0.2397
**PR status**	0.0983	0.3738
**HER2 status**	-0.0550	0.6189
**Ki67 expression**	0.0273	0.8052
**Histologic grade**	0.0376	0.7343
**Surgery type**	0.0749	0.5174
**Radiotherapy**	-0.0251	0.8282
**Chemotherapy**	0.0399	0.7305
**Immunotherapy**	-0.0658	0.5694
**Hormone therapy**	0.0850	0.4623

SDF-1α—stromal cell-derived factor 1α; ER—oestrogen receptor; PR—progesterone receptor; HER2—human epidermal growth factor receptor 2; Ki67—proliferation marker; significant differences are denoted by bold

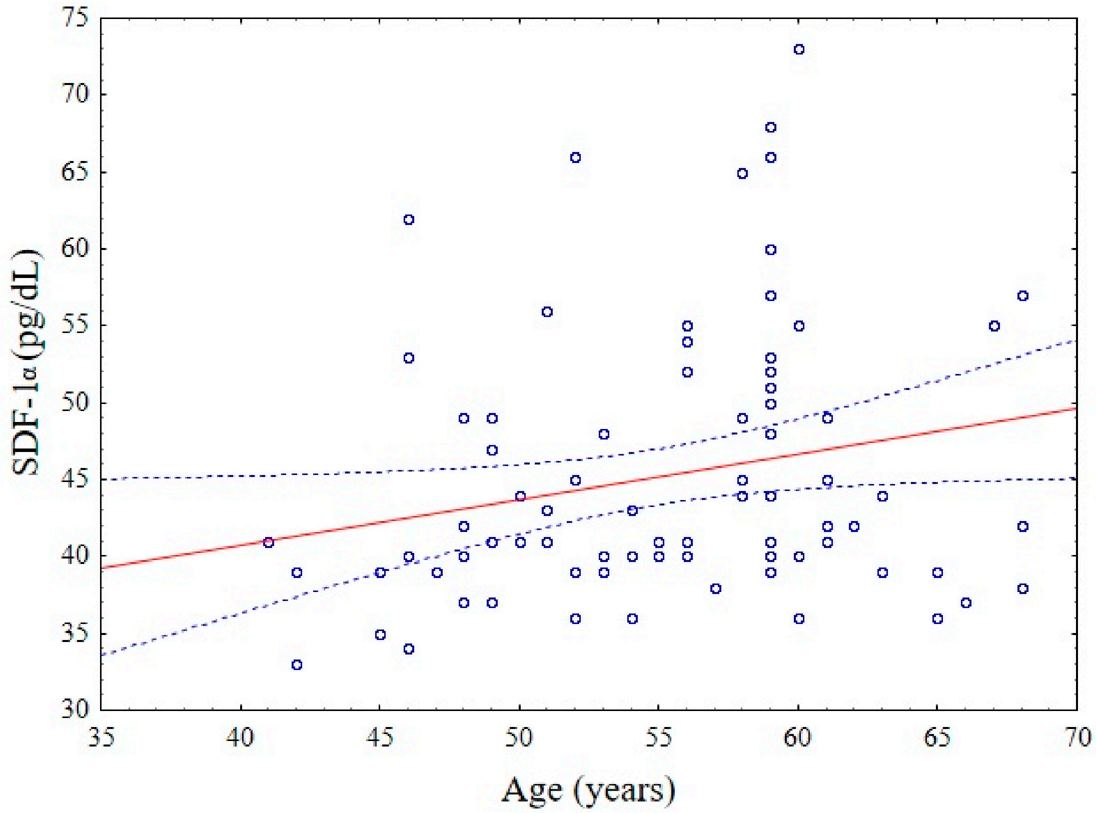

Figure S1. Scatter plot shows a positive correlation between a pre-treatment concentration of SDF-1α with patients' age (Spearman correlation coefficient, r = 0.2364; p = 0.0304).

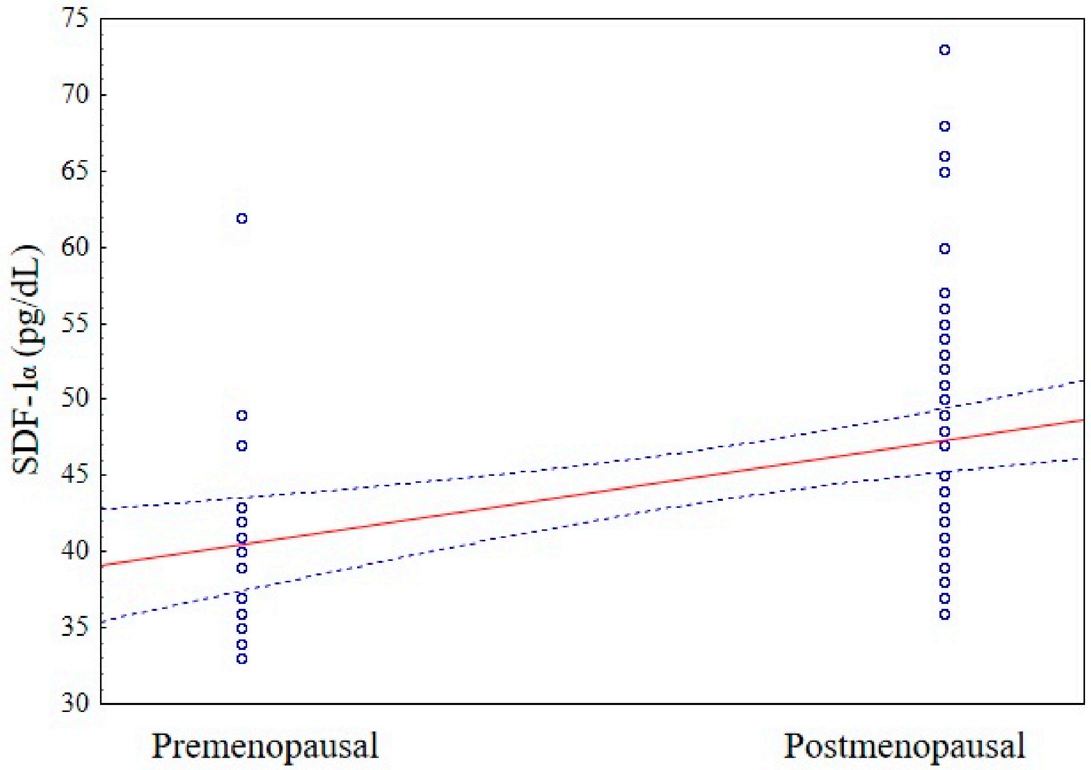

Figure S2. Scatter plot shows a positive correlation between a pre-treatment concentration of SDF-1α with patients' menopausal status (Spearman correlation coefficient, r = 0.4156; p = 0.0001).
